# Nano Gel/Hydrogel-Based Components for Battery Technology: An Overview

**DOI:** 10.3390/gels11090762

**Published:** 2025-09-22

**Authors:** Md Murshed Bhuyan, Kyungjun Lee

**Affiliations:** Department of Mechanical, Smart, and Industrial Engineering (Mechanical Engineering Major), Gachon University, 1342, Seongnam-daero, Sujeong-gu, Seongnam-si 13120, Gyeonggi-do, Republic of Korea

**Keywords:** nanogels, hydrogel, battery, electrolytes, electrodes, membranes

## Abstract

Battery technology represents a cornerstone in the evolution of the energy sector, driven by the urgent need for sustainable and efficient energy storage systems. Various materials, including metals, non-metals, semiconductors, and polymeric gel conductors comprise batteries, and their size and composition can significantly affect battery performance. The essential components of a battery are electrolytes, electrodes, nanogelators, and membranes that can be built up by using nanogels. The nanogel components significantly enhance the efficiency and stability of redox-active flow batteries, which makes them cheaper and eco-friendly. Little research has been conducted on nanogel-based battery technology. This study mainly focuses on the nanogels used in the components of batteries. The review explains the functions of nanogels in different electrolytes, electrodes, nanogelators, and membranes. This review explicitly discusses the current status and literature background of nanogels and hydrogels in battery technology. For anyone interested in delving deeper into the realm of nanogel-based batteries, this review article serves as a valuable resource, offering a thorough exploration of their role in revolutionizing modern energy storage systems.

## 1. Introduction

A solid three-dimensional polymeric network made of two or more components attached through covalent or noncovalent (physigel and chemigel) bonding and which can swell without dissolving in swelling media is referred to as gel/hydrogel [1]. Usually, polymeric hydrogels are polymer-solvent binary systems where the polymer retains solvent through H-bonding [2,3]. If the hydrogel can hold water thousands of times its dried weight, then the hydrogel is called superabsorbent [4]. Nanogels possess the properties of both hydrogels and nanomaterials. The size of nanogels ranges from 10 to 1000 nm, but the size 20 to 250 nm is considered standard. The first nanogels prepared from cholesterol-bearing pullulans were reported by Akiyoshi’s research group in 1993 [5,6]. Materials called conductive hydrogels, also known as gel polymer electrolytes (GPEs), are made to make ion transport easier. A polymer base, a salt, and a solvent or ionic liquid (IL) are the usual components of a GPE. The ionic liquid is trapped in the polymer matrix, the salt adds free ions to improve conductivity, and the solvent/IL dissolves the salt to function as a conducting medium. Together, these elements offer mechanical stability as well as an ion conduction medium [7,8]. Over other hydrogels and microgels, nanogels have some advantages, like a large surface area, enhanced functionalities, and an efficient application in electronics. Nanogels can be prepared from both natural polymers (proteins and polysaccharides) and synthetic polymers (poly(ethylene glycol) (PEG), polyethyleneimine (PEI), dendrimers, polypeptides, polyacrylates, polymethacrylates, poly(lactic acid) (PLA), poly(ε-caprolactone), and poly(lactic-co-glycollic acid) (PLGA)) [9]. The electrochemical cell can be defined as the electrochemical system where the chemical energy is converted into electrical energy through oxidation–reduction reactions that take place in the anode and cathode electrodes. The battery is a series of electrochemical cells connected with one another to supply the required electrical energy [10]. Conventional batteries are made of metal ions such as Li^+^, Zn^2+^, Na^+^, Mg^2+^, and Ca^2+^. Al^3+^ extracted from different mines resulted in the decline of resources. Extraction and processing of metal ions for battery manufacturing detrimentally affects our environment and ecosystem. The metal ions having large atomic radii, like K^+^, Al^3+^, and Zn^2+^, undergo phase changes during intercalation [11,12]. Non-metallic charges like ammonium (NH_4_^+^) and proton (H^+^) are used in batteries to improve the ionic conductivity [13]. Traditional metal-based batteries are not flexible, non-biocompatible, non-biodegradable, and rigid. To mitigate those limitations, a greener and safer alternative candidate, conductive hydrogel, can be taken into consideration and incorporated in manufacturing batteries. Recently, Abhishek Paudel et al. reported a full metal-free hydrogel-based battery where they used ammonium ion as a charge carrier, a polyaniline (PANI) anode, and a polypyrrole (PPy) cathode coupled with a quasi-solid-state (QSS) hydrogel electrolyte [14]. Nowadays, lithium, sodium, and zinc batteries are very popular. But the hydrogen evolution and dendrite growth limit their performance. Recently, Kento Kimura et al. reported a nanofiber hydrogel hydrogelelectrolyte where they used TEMPO-oxidized cellulose and found a stable Zn/electrolyte interphase [15]. Owing to the fast charge flow properties, the nanogel may make the battery more efficient. Nanogels are used as electrolytes, electrodes, and membranes, as shown in Table 1. Nanogel batteries employ an electrolyte that resembles silicone gel in both chemistry and appearance. The gel maintains the lead plates and their active material in place while improving the internal structural integrity of the battery. If the unit is shaken or vibrated, the gel “glues” onto the plates, essentially uniting the electrolytes and plates into a single piece that moves in unison. Owing to many benefits, gel batteries can be used in a variety of settings; for example, nanogel batteries do not need to be maintained. Even if the outside casing cracks, the likelihood of leakage is extremely low due to the large, viscous surface area and plasma-like characteristics of the electrolyte. As a result, gel batteries can be positioned at any angle. Nanogel batteries are also incredibly resistant to stress and vibration. Since there are no hydrogen emissions, one can charge them without worrying about keeping them in a ventilated space. Additionally, gel units have a greater discharge characteristic because of their improved deep cycling capabilities. Additionally, nanogel batteries may be readily recharged even after being depleted for an extended length of time. Despite the many benefits of nanogel batteries, there are some disadvantages, the first of which is cost. Nanogel batteries are far more expensive than traditional metal–acid batteries. Moreover, their rate of charging is quite slow. Furthermore, its charging must be stopped right after it is finished since it may cause holes in its electrolyte, which might lead to a reduction in charging capacity. Gel batteries must also be safeguarded against heat. Elevated temperatures might adversely affect the saturation and composition of the electrolyte. Due to their various advantages, gel batteries are appropriate for a diverse range of applications. The most prevalent examples encompass solar energy storage, ventilators in healthcare institutions, and arguably the most ubiquitous—electric vehicles [16,17,18,19]. The electrodes prepared from nanogels are less degradable, extend the lifetime of the battery, are biocompatible, and reduce environmental impacts [20,21].

Nanogels expand and absorb water as they swell, which raises the gel matrix’s free volume and ion mobility. By increasing the free volume and segmental mobility of polymer chains, moderate swelling can improve ionic conductivity and facilitate quicker transport of Li or Na. However, a higher water content might possibly reduce the electrochemical window, encouraging water electrolysis at lower voltages. In addition to decreasing mechanical integrity and raising the possibility of dendritic penetration, excessive swelling can dilute the polymer matrix [22]. For instance, a PVDF-HFP-based GPE that was compatible with high-voltage cathodes demonstrated a stable electrochemical window of about 4.6 V when swelling was managed [23]. The gel’s swelling state is linked to both mechanical and chemical stability. By enhancing electrode–electrolyte contact, controlled swelling can lower interfacial resistance. When the hydrogel is swollen, it becomes more hydrated, which can cause electrolyte leakage, hydrolytic breakdown of sensitive polymer backbones, and an acceleration of capacity decline [24]. To improve the performance of the battery, the hybrid nanogel electrolytes and electrodes may be researched and synthesized from the combination of inorganic and organic compounds in the future.

The objective of this review is to illustrate the function and efficiency of the electrolyte, electrode, nanogelator, and membrane prepared by using nanogel particles and subsequent application in different batteries.

**Table 1:** Nanogels/hydrogels in different components of battery.

Hydrogel	Preparation Method	Application Fields	Performance/Efficiency	References
TEMPO-grafted PNIPAM-co-APMA nanogels	A two-step synthetic method under mild aqueous conditions.	Redox flow battery electrolytes	Nanogels facilitate electron transfer between the electrode and nanogels in solution	[16]
Acetonitrile-based gel	A multi-step preparation process	Dye-sensitized solar cells	The largest η value of 4.17%	[25]
Imidazolium ionic liquid-based nanoparticle-integrated gel	Multi-step preparation method	Electrolyte of lithium-ion battery	Exhibits ionic conductivity of 3.2 × 10^−3^ S cm^−1^ at ambient temperature	[26]
3D graphene/PANI composite hydrogel	Free radical photopolymerization (UV-curing)	Electrode of Zn-ion hybrid cell	Hydrogel electrode exhibits a large capacity of 154 mA h g^−1^	[21]
Hemoglobin-poly (acrylic acid) nanogel	Multi-step preparation method	Bioelectrodes	Can retain >95% electroactivity after storing for 14 days	[20]
Poly(vinylidene fluoride-co-hexafluoropropylene) nanocomposite	Solution-cast technique	Sodium–sulfur batteries	Highest ion is 4.1 × 10^−3^ S cm^−1^ at room temperature	[27]
Copoly (phthalazinone biphenyl ether sulfone nanofiber–gel	A multi-step process involves electrospinning, infiltration, and in situ polymerization	Quasi-solid batteries, specifically lithium metal batteries	Ionic conductivity is 1.36 mS cm^−1^	[28]
Poly (vinylidene fluoride)-hexafluoropropylene nanosheet gel polymer electrolyte (GPE)	A multi-step preparation method involving precise hydrothermal synthesis	As electrolytes in lithium batteries	Batteries assembled with the Nano-GPE of show 128 mAhg^−1^ discharge capacities	[29]
Porous poly (vinyl alcohol) (PVA)-based nanocomposite gel	A phase-inversion method (a selective dissolution mechanism)	Electrolyte of zinc–air batteries	Shows ionic conductivity of 57.3 mS cm^−1^	[30]
Poly (vinylidene fluoride-co-hexafluoropropylene) based gel electrolyte	Multi step preparation method (ionic liquid-mediated nano-composite polymer gel electrolyte)	As electrolyte in rechargeable battery	Specific discharge capacity is 138 mAh/g	[31]

## 2. Battery Technology

**Figure 1:**
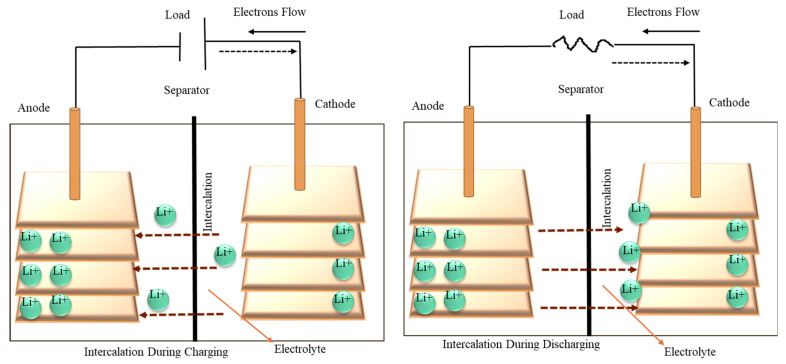
Intercalation reaction in lithium-ion battery: effect on cell characteristics.

In the 21st century, global industrialization has a great impact on electrical energy management and energy sources by their rapid utilization and development. Electrical energy and industrialization are proportionally dependent on each other, and no one can withstand it alone. On-demand electrical energy supply is an essential demand for the continuation of fulfilling the demands of the individuals. To supply a safe, reliable, and continuous supply of electrical energy, a massive electrical generation infrastructure has been built to commercially supply to the households and industrial sector globally [32]. The demand for electrical energy is increasing with population growth and with industrial development. For example, in the USA, it was reported that in 1950 the electrical energy expenditure was about 291 billion kWh, in the 1990s the consumption rose to around 3000 billion kWh, and in 2018 the amount was around 3946 billion kWh [33]. Electrical energy has a fixed supply system which can effectively generate electricity to a stationary place, but it cannot be used in mobile communication or in movement where the stored energy is the most convenient source of electrical energy to fill the gap. By industrial development, numerous products have been introduced in the last several decades, which mostly depend on stored energy, like cellphones, children’s toys, clocks, different electronic gadgets, laptops, and tablets. Those are run by battery power, which is stored energy. In medical technology, some inventions like pacemakers and insulin pumps implanted in the human body need a light-weight mobile power source. Moreover, in the most recent decade, to minimize carbon emissions and achieve sustainable development goals, the world is shifting the transportation system using electrical energy from fossil fuels, where the stored electrical energy plays an important role, like in electric vehicles, which are a growing industry in the modern world [32]. The battery can be defined as a collection of multiple connected electrochemical cells and a single working cell where it produces electrical energy through an oxidation–reduction process (redox), where oxidation causes one material to lose electrons while reduction causes another substance to gain electrons [34,35]. It is mentioned that the two processes do not necessarily happen in the same location, and without a corresponding reduction process, the oxidation cannot occur. Figure 1 illustrates a simple electrochemical cell where the chemical reaction takes place on the surface of cell electrodes. Redox-active materials or more electrochemically inert substances like platinum, mercury, gold, and graphite can make up these electrodes [36]. The oxidation develops at the anode, and at the same time, the reduction process takes place on the cathode side. To reduce polarization and allow the current flow, an ionic conductor as an electrolyte is necessary between the electrodes. A connection is made with both electrodes to complete the circuit by which it is allowing the battery to discharge and by which desired work is performed. The entire system needs to stay charge-neutral to keep working. Polarization occurred due to an accumulation of charge, which eventually stopped the electric current. The research on electricity in the early 1800s was mainly limited to what could be achieved through the collection and discharge of static electricity [37,38,39]. While it was possible to create high-voltage arcs, their practical use was constrained by low current and the extremely brief duration of discharge. Despite these limitations, the exploration of electrical phenomena ranged from attempts to split water via electrolysis to Benjamin Franklin’s famous lightning experiment and the early work of Luigi Galvani, known as Frog research [40,41,42,43]. In March 1800, Alessandro Volta, a professor of natural philosophy at the University of Pavia in Lombardy, Italy, invented a device capable of generating a continuous supply of electrical energy, which later came to be known as the “Voltaic Pile”. This invention significantly impacted the study of electricity. Due to the limited lifespan, electrode polarization, maintenance challenges, short discharge duration, user complexity, and corrosion issues of the metal disks, modifications were made by Cruickshank and Charles Wilkinson, who used a wooden separator instead of copper plates [44,45]. J. Frederic Daniell was credited with the invention of the “Daniell Cell”, which was successfully developed by Becquerel and Wach through their work on a two-fluid cell [37,46]. William Grove later advanced the Daniell Cell, and it was subsequently named the “Grove Cell” after replacing the carbon with an inert electrode material [37]. Both Grove’s and Daniell’s contributions laid the groundwork for the development of modern dry cell batteries. After extensive research, alkaline electrolytes were patented in France by Flix De Lalande in 1881 and in the USA by Georges Chaperson in 1883, although studies on alkaline solutions began earlier [47]. Further modifications and research led to patents granted in 1947 by Lewis Urry, Paul Marsal, and Karl Kordesh in Great Britain, followed by similar developments in the USA during the 1960s [48]. W.J. Sinsteden first described the lead–acid cell battery in 1854, and a few years later, Gaston Plante modified it into a more usable scale, though its performance remained limited [49]. Even in the 21st century, lead–acid cells continue to dominate the market, generating over $35 billion annually, primarily in the automotive sector [50]. However, their heavy weight and acidic electrolytes make lead–acid cells less suitable for portable applications. The introduction of nickel–cadmium batteries marked a significant advancement, starting with nickel–iron technology developed by E.W. Jungner in 1897, which was further refined by replacing iron with cadmium in 1901 [51,52]. Finally, the emergence of lithium-ion batteries has transformed the global energy storage market, as lithium exhibits higher activity and lower density than zinc, resulting in enhanced performance and a lighter weight. The lithium electrode potential was measured by Lewis and Frederick in 1913. The first commercial introduction of the lithium-ion battery occurred in 1991, attributed to Sony and Asahi Kasei. This innovation was established by Akira Yoshino, who created the first practical lithium-ion battery in 1985, building lithium cobalt oxide (LiCoO_2_) on earlier research conducted by John B. Goodenough and M. Stanley [53,54]. However, the lithium-ion battery generally used the intercalating electrodes [55] but besides the advantages, these electrodes have some disadvantages; for example, the intercalating reaction can be slower, which affects power output, repeated cycles can cause structural change and reduced efficiency, and some of them pose a risk to thermal stability [56,57,58]. The intercalating reaction can be defined from the recent study of the chemistry department of the Institute for Material Research at State University of New York as “a chemical reaction wherein lithium or hydrogen is inserted into a host matrix with essential retention of the crystal structure” [59]. Like the traditional battery, a lithium-ion battery consists of an anode, a cathode, and an electrolyte. However, the intercalation is a significant action in lithium-ion batteries. During the charging process, lithium ions transfer from the cathode to the anode, where they intercalate between the carbon layers of graphite, as demonstrated by Rachid Yazami in 1982 [60,61]. Lithium ions are electrochemically added to the graphite later while charging the battery. Lithium ions can fill up the voids with carbon atoms via this intercalation process, which effectively boosts the anode energy storage capacity. The anode materials undergo structural changes as lithium ions intercalate, causing volume expansion in the graphite layer, which is essential to prevent mechanical stress and degradation of the electrode materials [62,63]. Anode materials, electrolyte choice, and battery design impact intercalation efficiency. However, the removal of lithium ions from the anode, which is called dealloying, can create large volume changes, capacity loss and mechanical strain, which makes it a limited application for certain electrode materials. The intercalation process is improved by introducing new materials which can intensify the capacity and stability of LiBs. This entails investigating new substitute cathode materials and enhancing the electrochemical environment to facilitate the migration of lithium ions [60].

**Figure 2:**
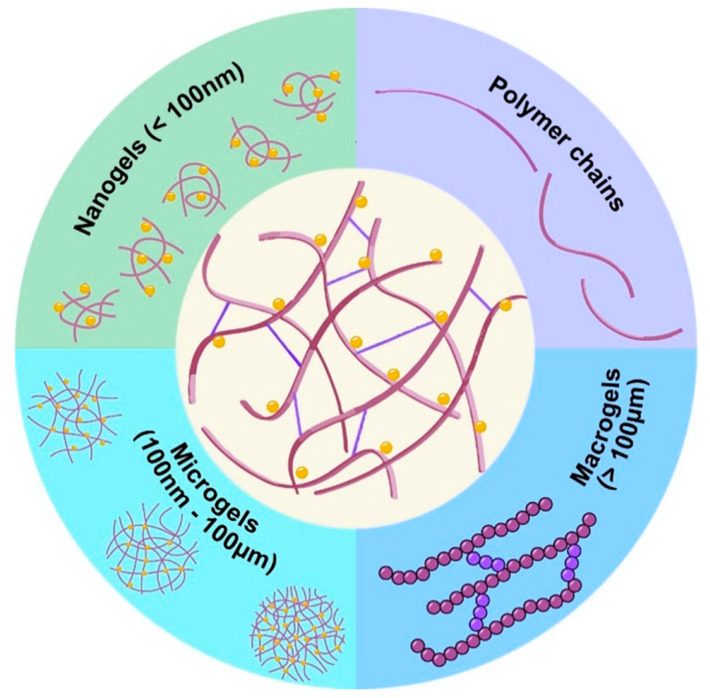
Nanogels and other gels of linear polymers [65].

Therefore, to consider those issues and to improve electrolyte performance and electrode stability, nanogels could be a great option for faster movement of lithium ions to improve the capacity of charge and discharge rate. Also, by using it, it is possible to increase the reduction in degradation, extend battery life, and stabilize the interface. Moreover, nanogels can help with battery heat management, which can make it safer by preventing the overheating issue [64]. When compared to linear polymers (Table 2), water-soluble macromolecular redox compounds such as nanogels have benefits in terms of stability, solubility, and viscosity. Figure 2 exhibits the visual demonstration of macrogels from linear polymers and nano/microgels. Linear polymer hydrogels are large, macroscopic networks that are perfect for local and structural uses (e.g., wound healing and tissue scaffolding). With their great responsiveness and targeting capability, nanogels are hydrogel particles at the nanoscale that are useful for precision drug delivery and nanomedicine. A step towards quicker, more regulated, and more focused therapeutic systems is represented by the transition from bulk hydrogels to nanogels [65].

**Figure 3:**
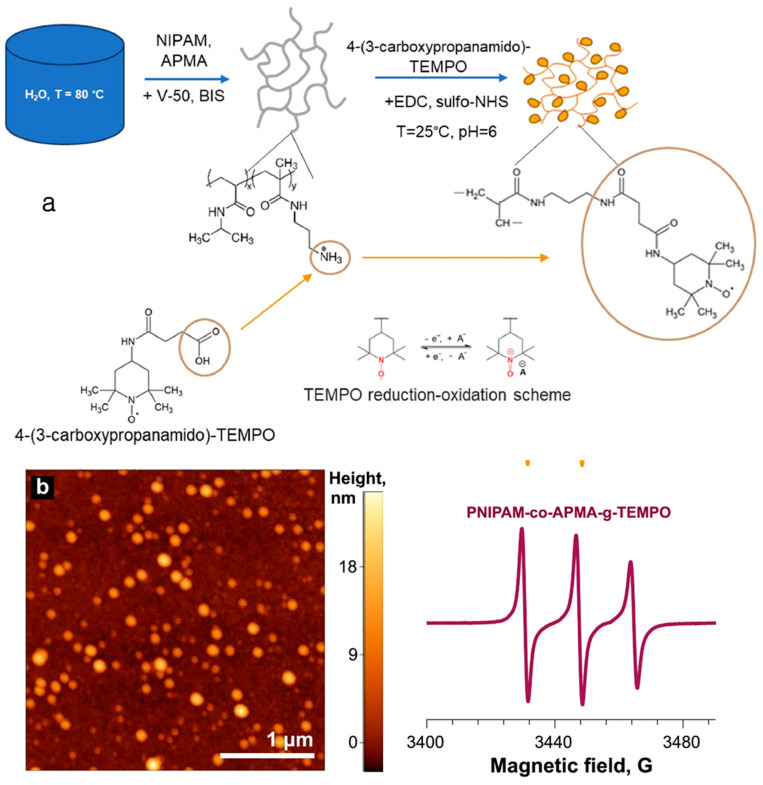
(**a**) Synthesis of PNIPAM-co-APMA-g-TEMPO nanogels; (**b**) AFM image and EPR spectra of PNIPAM-co-APMA nanogels (reused with copyright permission from ref. [16]).

In order to create nanosized cationic gels for application as redox flow battery electrolytes, Kozhunova et al. [16] grafted redox-active 4-(3-carboxypropanamido)-TEMPO units onto poly-(N isporopylacrylamide-co-N-(3-aminopropyl) methacrylamide hydrochloride), resulting in the formation of (PNIPAM-co-APMA)-g-TEMPO nanogels, shown in Figure 3a. Atomic force microscopy (AFM) and X-band electron paramagnetic resonance (EPR) spectra confirmed the nanogel formation shown in Figure 3b. AFM measurements showed a mean particle height of 20 nm and a diameter of 140 nm. EPR spectroscopy verified the existence and molar concentration of TEMPO functional groups. In this study, new TEMPO-grafted redox-active nanogels are presented along with a technique for determining the “effective” concentration and diffusion coefficient of TEMPO-groups inside the nanogel particles. Since the adsorbed nanogel layers mediate electron transport between electrodes and nanogels, dissolved TEMPO-grafted nanogels exhibit faster electron transfer.

Since the adsorbed nanogel layers mediate electron transport between electrodes and nanogels, dissolved TEMPO-grafted nanogels exhibit faster electron transfer. Therefore, PNIPAM-co-APMA-g-TEMPO nanogels have several benefits over earlier microgel versions, including a greater diffusion coefficient, simpler manufacturing, and less congested conditions for nitroxide groups, which improves potential electrode contact [16].

## 3. Nanogel Based Components of Battery

### 3.1. Nanogel-Based Electrolytes for Battery

**Figure 4:**
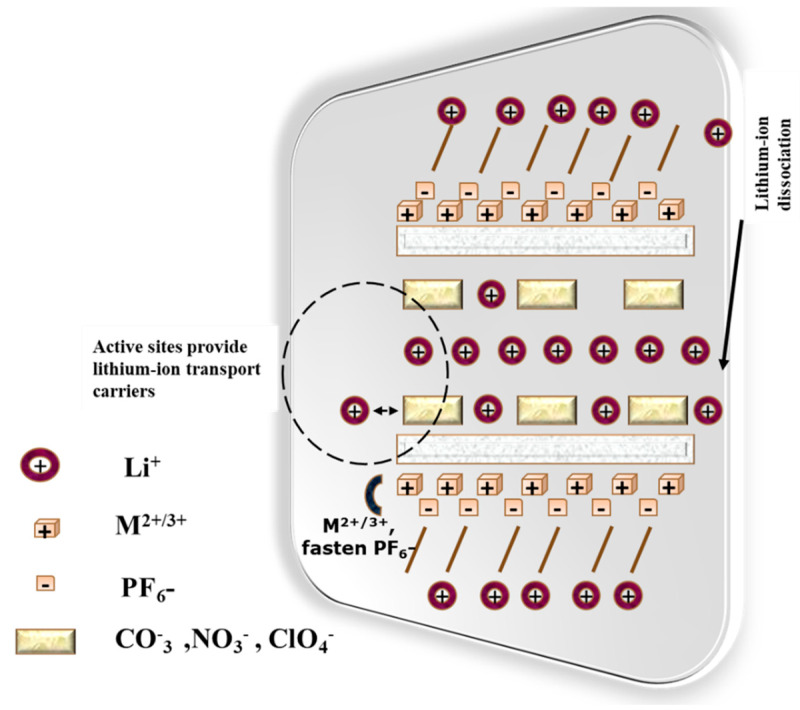
Ion exchange diagram for nanogel electrolyte in lithium-ion battery.

A battery electrolyte is a material which allows the movement of ions between the anode and cathode, enabling the electrochemical reactions necessary for energy storage and release. The electrolyte can be solid, liquid or gel, depending on the battery type. Gel electrolytes are a particular kind of electrolyte that possesses both liquid and solid characteristics [71]. In comparison to completely solid electrolytes, they are made to function better while overcoming the drawbacks of liquid electrolytes, such as evaporation and leakage [72]. Considering the applications and chemistry, the battery electrolyte has different types, like liquid electrolyte, solid polymer electrolyte, nanocomposite electrolyte, nanogel electrolyte, and molten salt electrolyte [73]. Among them, the nanogel electrolyte represents great advancement for future advancements in battery technology. The nanogel electrolyte is categorized as a special type of polymer electrolyte which mixes the properties of gel and composite electrolytes, showing significant performance in lithium-ion batteries. Due to the important key aspects, the nanogel electrolyte outperforms the conventional electrolyte types. Those aspects are higher ionic conductivity, improved stability, better mechanical strength, good thermal stability, and improved electrochemical performance. For example, gels that include BaTiO_3_ nanoparticles exhibit reduced conductivity and significantly more weight loss than the silicate nanogel [74,75]. The common examples of nanogel electrolytes are Li-fluorohectorite-based nanogel electrolytes, PVDF-HFP nanogel electrolytes, nanogel electrolytes with LiCF_3_SO_3_, and silica-based nanogel electrolytes. The nanogel electrolyte is responsible for executing several key roles in enhancing the performance and stability of batteries, like high ambient conductivity, mechanical robustness, metal electrode stability, reducing interfacial impedance, thermal stability, and flexibility. Wang et al. [29] reported the nanofiber gel electrolyte (Nano-GPE) composed of poly (vinylidene fluoride)-hexafluoropropylene copolymer exhibits excellent physical properties. To enhance the material’s characteristics, the modification involved the introduction of magnesium–aluminum-bilayered nanosheets (MgAl-LDs) integrated with different acidic ions. The Nano-GPE demonstrates outstanding properties, including an elongation at a break of 166% and a tensile strength of 6.95 MPa, indicating that the material is both strong and flexible—qualities that are essential for battery applications. For the stability and safety of lithium batteries, the electrochemical window of the electrolyte plays a crucial role; it has been reported to exceed 5 V. Additionally, it was found that the NO_3_^−^ ionic conductivity is elevated, which facilitates improved ion transport. Lithium batteries using the Nano-GPE modified by MgAl-LDs (NO_3_^−^) show enhanced performance across current densities ranging from 0.1 to 2 C. They achieve a discharge capacity of 155 mAh/g at 0.1 C and a reversible capacity of 135 mAh/g, while retaining 90% capacity after 100 cycles at 0.5 C, demonstrating remarkable cycling stability. Figure 4 illustrates the ion exchange diagram. Overall, the Nano-GPE exhibits promising performance and has potential applications in flexible and high-density solid-state batteries, addressing the growing demand for energy storage solutions across various sectors, particularly in portable and flexible electronics. Furthermore, it offers economic viability, providing an affordable option without sacrificing performance [29].

**Figure 5:**
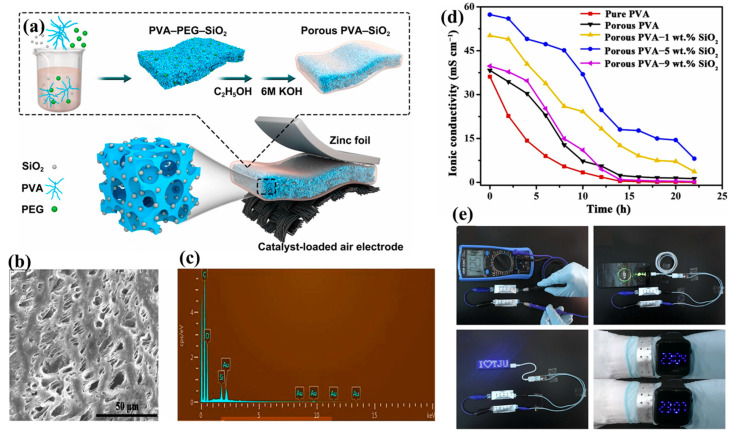
(**a**) Preparation procedure and inner structure of the porous PVA-based nanocomposite GPEs, flexible ZAB (**b**) FESEM image, (**c**) EDX analysis, (**d**) ionic conductivity as functions of time of pure PVA, porous PVA, and porous PVA–1, 5, 9 wt% SiO_2_ nanocomposite GPEs at 25 °C, and (**e**) an open circuit potential demonstration with two ZABs (reused with the permission from the reference [30]).

The flexible zinc–air batteries demonstrate a promising energy source in recent decades due to their low cost, environmental friendliness, and high theoretical energy density (1086 Wh kg^−1^) [76,77,78,79]. Flexible electronics require zinc–air batteries (ZABs) because of their high energy density and affordability. Gel polymer electrolytes (GPEs), which provide an ionic connection between the cathode and anode, are essential in ZABs. Poor electrolyte retention capabilities and low ionic conductivity are two drawbacks of traditional polyvinyl alcohol (PVA)-KOH GPEs. To mitigate the problems, Fan et al. [30] developed a novel porous PVA-based nanocomposite gel polymer electrolyte (GPE) containing silica (SiO_2_) fillers for flexible zinc–air batteries (Figure 5a).

A field-emission scanning electron microscopy equipped with energy-dispersive X-ray (EDX) revealed the microstructure and composition of the electrolyte shown in Figure 5b,c. The ionic conductivity vs. time curve for pure PVA, porous PVA, and PVA-SiO_2_ nanocomposite gel with different SiO_2_ percentages is shown in Figure 5d. Superior electrolyte retention capacity and remarkable ionic conductivity (57.3 mS cm^−1^) were demonstrated by the optimized porous PVA-based nanocomposite GPE with 5% SiO_2_. High power output, steady discharge performance, and exceptional cycling stability over 48 h were all displayed by the ZAB with this electrolyte. The flexible ZAB sets demonstrated exceptional flexibility without performance degradation even under the various bending conditions shown in Figure 5e, powering a variety of electronic devices such as mobile phones, electric fans, and LED screens [30].

### 3.2. Nanogel-Based Electrodes for Battery

**Figure 6:**
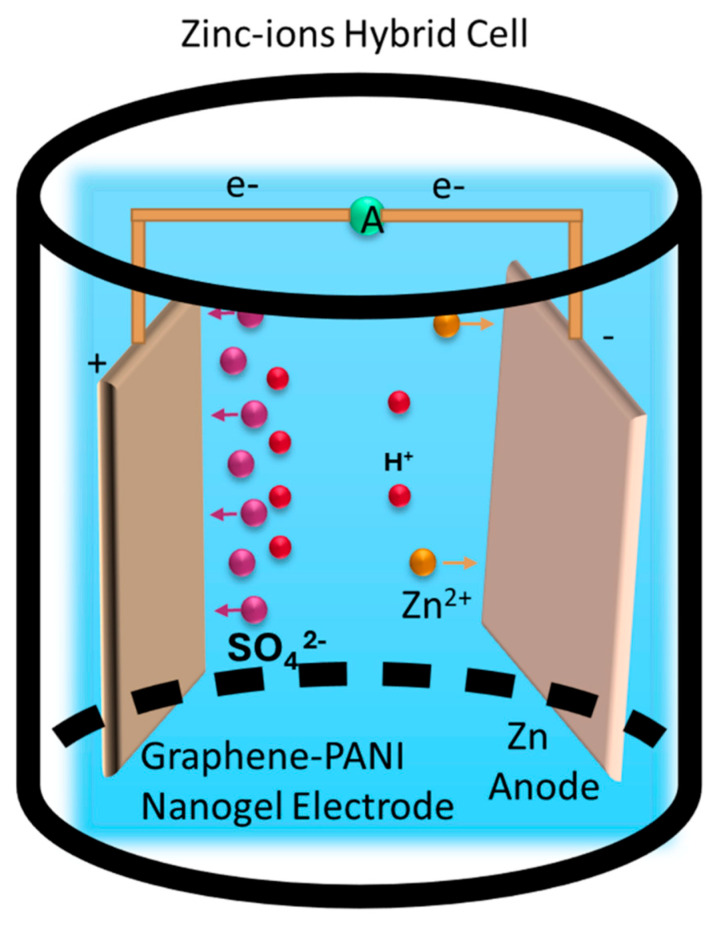
Three-dimensional graphene-PANI nanogel electrode in battery.

Nanogel electrodes consist of nanogel materials of nanoscale size, leading to enhanced conductivity and functionalities. These electrodes are frequently used in metal (example: Li^+^, Zn^2+^, Na^+^) ion batteries, supercapacitors, biomedical devices and soft electronics [80,81]. Jianwei et al. developed a zinc-ion hybrid cell by using a graphene and polyaniline (GP-PANI)-conducting polymer composite nanogel as an electrode. The Zn-ion hybrid cell (ZiHC) presents promising features to address these needs. To enhance its electrochemical performance, the ZiHC requires careful integration of materials and design concepts. It is structured with a capacitor-type cathode and a multivalent metal battery-type anode, specifically utilizing zinc (Zn) as the anode material shown in Figure 6. This combination capitalizes on the benefits of both capacitor and battery technologies, aiming to achieve higher power and energy density. A significant innovation in this development is the use of a 3D graphene-PANI composite hydrogel as the cathode. This material features an intelligently engineered three-dimensional (3D) nano-architecture, which increases the active interface between the electrode and electrolyte. This design is expected to enhance the overall capacity and efficiency of the ZiHC [82,83].

The assembly of the Zn-ion hybrid cell involves a hydrogel film pre-saturated with a 2 mol L^−1^ ZnSO_4_ solution [84]. Moreover, the electrochemical performance of the ZiHC is characterized by a higher specific capacity of 154 mAh g^−1^, good rate performance, and excellent capacity retention of 80.5% after 6000 charge–discharge cycles [85,86]. These results indicate that the 3D graphene-PANI composite hydrogel cathode significantly enhances the capacity of the ZiHC compared to conventional electrical double layer (EDL) cathode materials. Incorporating a capacitor-type cathode with a battery-type anode in the ZiHC represents a notable advancement in energy storage technology. Additionally, the innovative application of a composite hydrogel electrode establishes the ZiHC as a promising option for next-generation energy storage devices [87].

Ghimire et al. [20] reported hemoglobin-poly (acrylic acid) nanogel bioelectrodes that are stable at high temperature along with better electrochemical performance. The construction of hemoglobin-poly (acrylic acid) (Hb-PAA) nanogel bioelectrodes involves several key steps and characteristics that enhance their stability and electroactivity. Random conjugation with high-molecular-weight poly (acrylic acid) (PAA, MW 450K) chemically modifies the Hb by wrapping met-hemoglobin (Hb) in the polymer. This procedure stabilizes it and produces a nanogel structure that retains Hb’s functional properties by improving its stability under a variety of conditions [20]. The distinct shape of the nanogels increases adherence to the electrode surface; moreover, the performance of the bioelectrodes depends on the enhanced electron transfer rates made possible by this morphology. When exposed to high temperatures, such as 122 °C during steam sterilization, the Hb-PAA nanogels can withstand these conditions without compromising their structural integrity or peroxidase-like properties, which are essential for sterilization purposes. In cyclic voltammetry (CV), the bioelectrodes manufactured from Hb-PAA nanogels exhibit distinct quasi-reversible redox peaks for efficient electron transfer. The stability of the electrode is reflected in the fact that they maintain more than 95% of their electroactivity after 14 days of ambient temperature storage. Among the Hb-based biosensors, the Hb-PAA nanogel enables the bioelectrode to achieve an excellent capability for detecting hydrogen peroxide, with a detection limit of 0.5 µM. This capability plays a vital role in facilitating effective communication between the Hb redox center and the underlying electrode. Since the process for producing these bioelectrodes is modular, they can be constructed to work with different proteins or enzymes that have appropriate ligation sites. This adaptability enables the development of a variety of bioelectrodes with different functions [57,58]. However, the formation of Hb-PAA nanogel bioelectrodes is a sophisticated process that combines chemical modification, nanogel morphologies, and electrochemical optimization to create stable and effective biosensors. By providing hemoglobin with a stable, protected environment, facilitating direct electron transfer, enhancing electroactivity, and preserving thermal stability, the hemoglobin-poly (acrylic acid) nanogels function effectively as bioelectrodes.

### 3.3. Nanogel-Based Gelators in Battery

**Figure 7:**
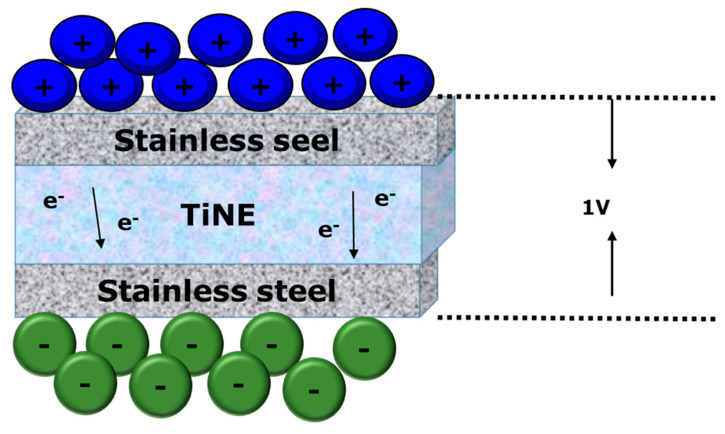
Ti-nanogelator electrolyte (TiNE) for battery.

A material which can create a gel-like structure at the nanoscale is defined as nanogilator and usually comprises nanoparticles with a porous matrix that can retain liquids, including ionic liquids, in place. These properties enable nanogelators to enhance electrolytes and develop their efficiency and stability in a range of applications, for example, medication systems and batteries. Nanogelators intend to increase system efficiency and safety by offering a stable path for ion transport. As they can change their structure according to the ionic liquids being utilized because of their characteristics like pore size and ionic conductivity, the adaptability is significant for the energy storage and delivery system. Whereas with the incorporation of titanium oxide (TiO_2_) as a primary component, the nanogelator formed as a specific type of Ti-nanogelator. These characteristics greatly lower the possibility of fire or explosion, which is a problem of the traditional liquid electrolyte used in lithium batteries. It can form a porous structure which can bind ionic liquids and form a solid electrolyte for the lithium-ion battery. The porous structure, nonflammability, and self-regulation are advantageous properties of Ti-based nanogelators [88,89]. The formation of Ti-nanogelator follows a specific synthesis process where the ionic liquid and titanium-based matrix are involved. In the synthesis process, the ionic liquid (IL) serves as an electrolyte component where the (IL) holds the cations and anions that are significant for nanogel electrochemical properties. During the formation of the nanogelator, the ionic liquid acts as a template solvent. Ionic liquid molecules are integrated into the titanium nanogel’s matrix through self-assembly, creating stable composite materials as they arrange within the titanium framework, as shown in Figure 7. The Ti-nanogelator matrix has a porous structure with particle sizes ranging from 5 to 60 nm, providing channels that enhance ion transport within the electrolyte, which is important for battery performance [88]. The Ti-nanogelator demonstrates different phases depending on their molar ratio of the ionic liquid to the nanogelator. However, the Ti-nanogelator pore size can also be adjusted through the formation process. The nanogelator matrix average pore diameter can be changed to better transport ions as the size of the cations in the ionic liquid grows [90]. Therefore, by using ionic liquids to self-assemble, the Ti-nanogelator creates a stable, porous structure which is ideal for ion transport, making it appropriate for use in lithium batteries.

### 3.4. Nanogel-Based Membranes for Battery

The membrane is a thin, flexible layer that acts as a barrier, allowing certain substances to pass through while blocking others. In battery technology, a membrane serves as a separator placed between the anode and cathode, referred to as a permeable membrane. Its primary function is to prevent direct contact between the two electrodes, which helps to avoid short circuits, while still permitting ionic charge to flow. This design enhances the efficiency of ion movement during the charging and discharging processes. Additionally, according to the National Renewable Energy Laboratory (NREL), the membrane plays a critical role in battery performance, safety, longevity, and maintaining electrochemical stability. It also helps to increase energy density and prevent thermal runaway. A study conducted by the University of California predicts that advancements in separator technology will improve battery performance by up to 30% by 2030. This enhancement is expected to significantly impact various devices, including automobiles, cell phones, and other electronic devices that rely on batteries for storage energy [91]. There are different types of separators used in batteries, as mentioned in Table 3, with advantages and disadvantages.

**Figure 8:**
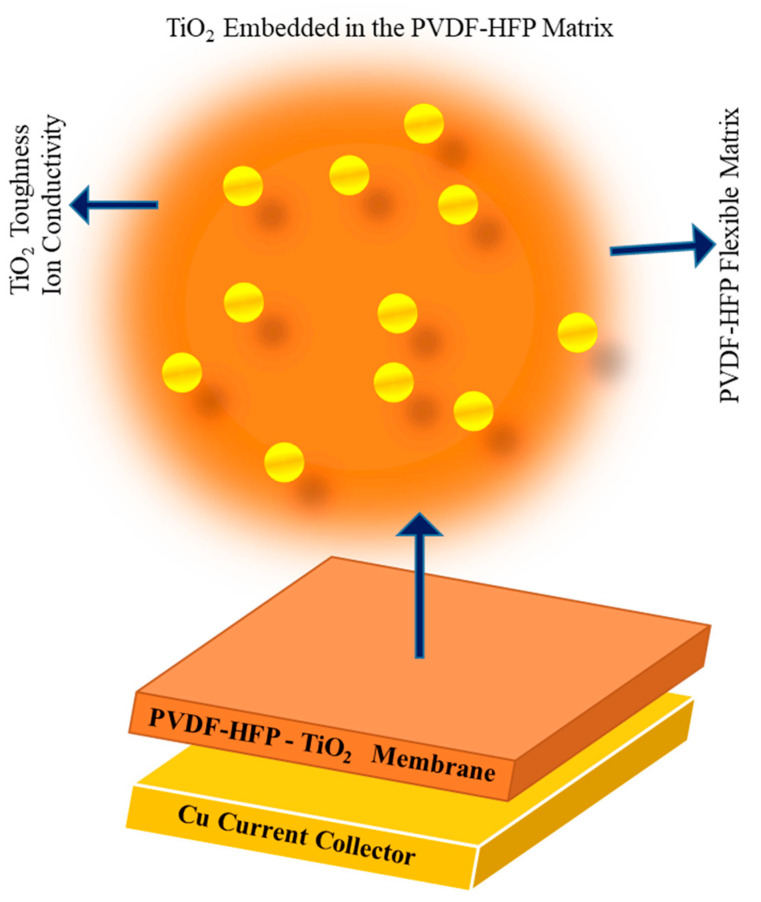
PVDF-HFP-TiO_2_ hybrid membrane.

To address the issues, the nanogel membrane presents a better option due to its highly porous structure and efficient ionic transport compared to polymer-based and nano-woven fabric separators. In comparison to ceramic-coated separators, it offers excellent thermal stability and maintains mechanical integrity under stress, outperforming glass fiber separators. The ability to customize the nanogel membrane to meet specific battery requirements is a significant advantage. However, due to the complexity of its manufacturing process, composite separators may be less suitable. Additionally, unlike other polymer-based separators that may degrade over time, the nanogel membrane resists chemical degradation and provides long-term performance [99,100]. Furthermore, nanogel membranes have become a game-changing energy storage technology, providing distinct benefits for lithium-ion, solid-state, and flow batteries. Their suitability in various applications, for example, consumer electronics, electric vehicles, and renewable energy storage, is because of their applicability and exceptional technical qualities like conductivity, stability and flexibility. In lithium-ion batteries, the nanogel membrane is widely used to enhance mechanical stability and ionic conductivity, where the membrane acts as an electrolyte or separator which prevents dendrite formation as well as improves its safety [101]. Such as gel polymer electrolytes (GPEs), based on poly(ethylene oxide) and lithium bis(trifluoromethylsulfonyl)imide (LiTFSI), have been identified with high ionic conductivity (up to 4.8 mS/cm), which makes them ideal for LiBs [102]. In addition, nanogel membranes are significant in overcoming the drawbacks of conventional solid electrolytes in solid-state batteries. Stable and high-performing solid-state batteries are made possible by composite solid-state electrolytes (CSEs) that incorporate nanofibers, for example, lithium superionic conductive nanofibers, which have demonstrated ultrahigh ionic conductivity (12.6 mS/cm) and a wide voltage window (5.2 V) [103,104]. Another less commonly used type of nanogel membrane in flow batteries is due to its separator efficiency and ion transport. In a flow battery system, issues with ion crossover and electrolyte stability may be resolved by their high porosity and mechanical strength [105]. The nanogel membrane shows high ionic conductivity because of its porous structure and the incorporation of ionic liquids or lithium salts. For instance, due to the increased mobility of Li^+^ ions, membranes made of polyacrylonitrile (PAN) nanofibers containing lithium salts have demonstrated ionic conductivity as high as 9.0 × 10^−5^ S/cm [106]. The key advantage of the nanogel membrane is its stability. Inorganic fillers, like TiO_2_ nanoparticles, have been used in composite electrolytes to increase their thermal stability and resistance to chemical degradation. These membranes function efficiently and have a broad temperature range (−40 to 100 °C) [101,107]. For flexible energy storage devices, the nanogel membranes are highly flexible and mechanically robust. For example, electrolytes reinforced with cellulose nanofibrils have shown tensile strengths of up to 9.66 MPa, which ensures longevity in demanding applications [102,108]. However, for lithium layer growth, control mechanisms of the inorganic monohybrid membrane are presented in Figure 8.

**Figure 9:**
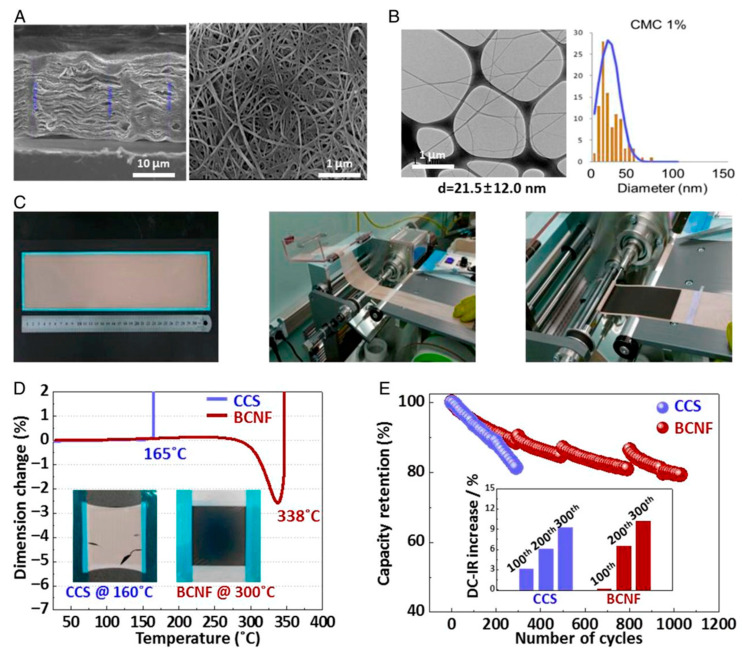
Bacterial cellulose nanofiber (BCNF) membrane and battery performances. (**A**) Scanning electron microscopy (SEM) depicts the BCNF membrane in both side (left) and top (right) views. (**B**) BCNF is made with 1% carboxymethylcellulose (CMC) and its diameter distribution using transmission electron microscopy (TEM). (**C**) large-area BCNF membrane and roll-to-roll production of a cylindrical LIB complete cell. (**D**) Thermomechanical analysis (TMA) of the BCNF and ceramic-coated separators (CCS). The BCNF demonstrated a significantly greater thermal tolerance up to 338 °C, higher than the CCS, which had a tolerance of 165 °C. The thermal resistance in a fixed frame at 160 °C (CCS) and 300 °C (BCNF) is displayed in the inset. (**E**) CCS and BCNF life cycle performances in entire cells of 18,650 batteries. After every 100 cycles at 25 °C, the relative direct current–internal resistance (DC-IR)-increasing ratios are displayed in the inset (Reused from reference [113]).

To control the surface growth of lithium layers and enhance the electrochemical performance in lithium metal batteries, the inorganic monohybrid membrane composed of PVDF-HF or TiO_2_ plays a significant role. By incorporating lithiophilic materials that promote even nucleation and lithium development during electrochemical processes, the hybrid membrane facilitates homogeneous lithium deposition. This uniformity is crucial for preventing lithium dendrite formation, thereby reducing the risk of battery failure and safety issues [109,110,111]. TiO_2_ was utilized as an inorganic material as a polymer. A very flexible polymer, PVDF-HF serves as the matrix for the inorganic compounds. The protective layer, designed from PVDF-HF or TiO_2_, not only increases mechanical strength but also enhances ion conductivity. Furthermore, the mechanical strength developed during the charging and discharging cycles suppresses dendrite growth. The incorporation of TiO_2_ nanoparticles into the PVDF-HFP matrix significantly increases the ion conductivity, achieved through Lewis’ acid–base interactions among the polymer and lithium salt ions, which contribute to improved electrochemical performance. Electrochemical measurements of the hybrid membranes demonstrate low overpotentials of less than 20 mV and a higher Coulombic efficiency of 80% over one hundred cycles. The inorganic composites enhance both mechanical characteristics and ion conductivity, leading to this impressive performance. Additionally, the surface of the PVDF-HFP/TiO_2_ hybrid membrane exhibits minimal roughness, which is favorable for homogeneous lithium deposition, and the measured surface roughness is confirmed to be below 100 nm, indicating a well-formed protective layer [112]. So, the inorganic nanohybrid membrane promotes uniform deposition, mechanically suppresses dendrite formation, and increases ion conductivity to control lithium layer surficial growth and improve lithium metal battery electrochemical performance. A safe and sustainable material alternative for large-scale batteries is the bacterial cellulose nanofiber (BCNF) membrane. Battery full cells using the BCNF separator were shown by Gwon et al. [113] to exhibit exceptional cycle stability, retaining 80% of its capacity even after 1000 cycles. By optimizing metabolic flux, which attempts to substitute BCNF with chemical-derived separators, researchers were able to increase BCNF output by 31.5%, raising worries about safety and the environment. Figure 9 displays the BCNF membranes’ characterization and performance as a battery membrane, demonstrating their strong 3D network structure, high thermal stability, porosity, and hydrophilicity. Their characteristics guarantee mechanical strength and thermal stability while facilitating quick Li ion transport in lithium-ion batteries (LIBs) [113].

## 4. Scope of Improvement

For future research, a key aspect of nanogels’ performance is their ability to withstand repeated cycles of charging and discharging. While some studies have exhibited promising results, further investigation is necessary [56,57]. One of the biggest challenges in nanotechnology and pharmaceutical manufacturing is scaling up the manufacture of nanogel to the kilogram level while maintaining nanoscale homogeneity. Nonetheless, recent developments have produced encouraging outcomes, especially in microfluidic systems [114]. It is technically possible to scale up nanogel synthesis to kilogram-level manufacturing while maintaining nanoscale homogeneity, but only in continuous or well-optimized batch systems with meticulously designed procedures that regulate mixing, kinetics, and crosslinking. The most reliable methods employ emulsion/precipitation polymerizations in stirred tanks where nucleation and growth are strictly regulated, or continuous flow (micro/milli-reactors) with numbering-up [81,115]. The interfacial resistance between the nanogel and electrode materials can significantly reduce the performance of nanogels in battery applications, ultimately lowering the overall efficiency of the battery. To address these challenges, it is crucial to develop methods that reduce resistance and enhance interfacial compatibility [58,59]. The Ti-nanogelator solid electrolyte presents a promising avenue, particularly for improving electrolyte properties such as cycling stability and interfacial compatibility. A better understanding of the reaction mechanisms between the electrode and the Ti-nanogelator solid electrolyte at elevated temperatures could lead to the development of more efficient electrodes capable of operating under diverse conditions. Additionally, for commercial applications in various energy storage systems, including lithium and sodium-ion batteries, optimizing production methods is essential. This optimization would aim to reduce production costs and ensure scalability, making these materials more feasible for widespread use in the energy sector [53].

## 5. Conclusions

The introduction and the basic knowledge on nanogels and their application in the parts of battery electrolyte, electrode, nanogelator and membrane have been depicted in this review. The nanogel electrolytes are made of metal, silica, conductive polymers, and ionic salt solutions, accompanied with the respective electrodes and membrane. Most of the nanogel electrode compositions belong to conductive polymers such as PANi, PPy, PVP, etc., which improve its conductivity and stretchability. Nanogelators are vital auxiliary materials incorporated in batteries to enhance efficiency. Hence, titanium and ionic liquid are the best choices for the preparation of the nanogelator. Although nanogel materials have revolutionary advantages, including increased efficiency and environmentally favorable qualities, there are drawbacks to using them in batteries. With their improved mechanical stability, strong ionic conductivity, and special qualities like flexibility and self-healing, nanogel and hydrogel components have great potential for the future of battery technology. Among the drawbacks are possible problems with cost-effectiveness, long-term stability, and scalability. Targeted research is needed to address these shortcomings to create novel ways to go beyond current obstacles. For example, innovations may result from better material combinations, improved nanogel production methods, and more thorough research into how these materials interact in battery systems. The main perspective is that batteries will become safer, more efficient, and more eco-friendly, especially for wearable and flexible applications. This paper outlines the importance of comprehending these constraints as a first step towards developing reliable and sustainable energy storage systems driven by nanogels, paving the way for further research and advancement in the area.

## Figures and Tables

**Table 2:** Differences between conventional hydrogels and nanogels.

Feature	Conventional Hydrogels	Nanogels	References
Structural difference	Bulk gel networks of natural or synthetic linear polymer chains that are macroscopic (mm–cm size). Network is crosslinked either physically (H-bonding, ionic, hydrophobic) or chemically (covalent). Greater capacity to retain water due to its larger mesh size.	Particles at the nanoscale, usually between 20 and 200 nm, are scattered across medium. Network of crosslinked polymers restricted to nanoscale regions. Chemical crosslinking is frequently used, however for fine control, self-assembly or microfluidic synthesis are alternative options. High surface-to-volume ratio; smaller mesh size.	[66,67]
Physical and Chemical Properties	Tissue-like, soft qualities. ballooning in bulk; a delayed reaction to stimuli.	Nanoscale particles contain water. The tiny size and large surface area causes rapid swelling and deswelling. very adjustable in terms of temperature, redox, pH, and enzyme triggers.	[68]
Functional Difference	Drugs scattered over the bulk gel; mostly released via diffusion. Serve as a reservoir or scaffold. Tissue scaffolds, contact lenses, and wound treatments.	The release of drugs enclosed in nanogel particles may be targeted and stimulated. Systemic circulation, tailored delivery (such as tumor targeting), and injectable. Serves in intracellular imaging, gene therapy, targeted drug delivery, and nanomedicine.	[65],
Advantages and Limitations	High water retention, biocompatibility, ease of synthesis, and suitability for large-scale uses. Poor mechanical strength, slow reaction time, and restricted ability to aim.	High surface area, injectable, focused delivery, and adjustable response. More intricate production, problems with stability in vivo, and possible immune system clearance.	[65,69]
Application in Battery	For flexible or stretchy batteries, their bulk structure offers strong mechanical support. Ionic conductivity is facilitated by high-water content, particularly in aqueous battery systems.	They improve charge/discharge rates by facilitating quicker ion diffusion due to their large surface area and porosity. Their tiny size and dispersibility make them easy to include into composite electrodes or electrolytes.	[70]

**Table 3:** Different types of membranes used in battery.

Separator Name	Advantages	Disadvantages	Reference
**Polymer-Based Separator**	It is widely used in lithium ion batteries due to their chemical stability, light weight, flexibility, and cost-effectiveness.	Compared to the other types, it has limited thermal stability and mechanical strength.	[92,93]
**Ceramic Coated Separator**	Mechanical strength and thermal efficiency are higher, which increases safety and lowers the risk of thermal runaway.	Potential brittleness and production cost are higher.	[94]
**Glass Fiber Separator**	It has improved ionic conductivity because of good chemical stability and high porosity.	For higher stress applications, it is unsuitable because of its fragility and low durability.	[95]
**Nano-woven Fabric Separator**	It is often used in lead–acid batteries due to its better mechanical strength and flexibility.	Compared to a polymer-based separator, it has low ionic conductivity.	[96,97]
**Composite Separator**	It combines the advantages of several materials to provide improved durability and performance.	The manufacturing process is complex and has a higher cost.	[98]

## Data Availability

Data is contained within the article. Further inquiries can be directed to the corresponding authors.
